# The Effects and Mechanisms of Sennoside A on Inducing Cytotoxicity, Apoptosis, and Inhibiting Metastasis in Human Chondrosarcoma Cells

**DOI:** 10.1155/2022/8063497

**Published:** 2022-08-31

**Authors:** Jiamei Le, Houlin Ji, Peixian Pi, Kaijie Chen, Xuefeng Gu, Yujie Ma, Yi Fu, Yongning Sun, Xiaoxiao Zhou, Hailong Wu

**Affiliations:** ^1^Shanghai University of Medicine & Health Sciences Affiliated Zhoupu Hospital, Shanghai, China; ^2^Shanghai Key Laboratory of Molecular Imaging, Collaborative Innovation Center for Biomedicine, Shanghai University of Medicine & Health Sciences, Shanghai, China; ^3^Shanghai University of Traditional Chinese Medicine, Shanghai, China; ^4^School of Health Science and Engineering, University of Shanghai for Science and Technology, Shanghai, China; ^5^School of Pharmacy, Shanghai University of Medicine and Health Sciences, Shanghai, China; ^6^Department of Cardiology, Shanghai Municipal Hospital of Traditional Chinese Medicine, Shanghai University of Traditional Chinese Medicine, Shanghai, China

## Abstract

Currently, developing therapeutic strategies for chondrosarcoma (CS) remains important. Sennoside A (SA), a dianthrone glycoside from Senna and Rhubarb, is widely used as an irritant laxative, weight-loss agent, or dietary supplement, which possesses various bioactive properties such as laxative, antiobesity, and hypoglycemic activities. For the first time, our results suggested that cell proliferation and metastasis were inhibited by SA in CS SW1353 cells. SA induced cell growth arrest by inhibiting cell proliferation. The changes of N-cadherin and E-cadherin levels, the markers associated with epithelial mesenchymal transition (EMT), suggested the EMT-related mechanism of SA in inhibiting cell metastasis. Besides, SA significantly stimulated apoptosis in CS SW1353 cells, leading to cell death. The increase of Bax/Bcl2 ratio confirmed that the internal mitochondrial pathway of apoptosis was regulated by SA. In addition, the prediction of network pharmacology analysis suggested that the possible pathways of SA treatment for CS included the Wnt signaling pathway. Notably, the protein levels of the components in the Wnt pathway, such as Wnt3a, *β*-catenin, and c-Myc, were downregulated by SA in CS SW1353 cells. To sum up, these results demonstrated that the suppression of the growth, metastasis and the stimulation of cytotoxicity, and apoptosis mediated by SA in CS SW1353 cells were possibly caused by the inhibition of the Wnt/*β*-catenin pathway, indicating an underlying therapeutic prospect of SA for chondrosarcoma.

## 1. Introduction

Chondrosarcoma (CS), the second most common bone tumor, is a cartilage-forming malignant tumor accounting for approximately 30% of bone tumors [1, 2]. The mainstay treatment options for patients with CS include surgery, chemotherapy, and radiation therapy. However, the insensitivity and poor response to conventional chemoradiotherapy generate serious challenges to CS [3, 4]. Recent studies have explored the curative effect of new therapies from Traditional Chinese Medicine (TCM) for CS, such as resveratrol, baicalin, and andrographolide [5–7]. Consequently, advances in research findings, especially from TCM, may provide us with more effective treatment modalities for CS.

Sennoside A (SA), a dianthrone glycoside, is a natural compound derived from the medicinal parts of TCM, such as the leaves of Senna and the roots of Rhubarb. SA has been widely used as an irritant laxative, weight-loss agent, or dietary supplement, with a variety of beneficial effects, such as laxative, antiobesity, hypoglycemic, hepatoprotective, anti-inﬂammatory, and anticancer activities [8]. So far, SA has been reported as a potential agent against pancreatic cancer and hepatocellular carcinoma (HCC). SA was identified as an effective inhibitor of slingshot homologs to weaken actin dynamics by blocking dephosphorylation of phosphor-coﬁlin, thereby inhibiting metastasis in pancreatic cancer cells [9]. Moreover, SA has been found to suppress HCC metastasis, possibly by inhibiting major tumor-related pathways, such as tumor necrosis factor, nuclear factor-kappaB, and vascular endothelial growth factor (VEGF) pathways [10]. However, it is currently unknown whether SA is effective against the human chondrosarcoma cell, as well as the molecular mechanisms behind these effects.

The study of network pharmacology is a powerful research approach to predict the underlying mechanisms of drug treatment for diseases based on existing databases, which can facilitate drug development. In our study, the results of network pharmacology analysis and validation experiments in vitro confirmed that SA reduced cell viability, metastasis, and stimulated cytotoxicity, apoptosis in chondrosarcoma SW1353 cells, due to the inhibition of the Wnt/*β*-catenin pathway. These results revealed the underlying molecular mechanism of SA-mediated anticancer effects in chondrosarcoma SW1353 cells, which might provide an insight into new therapeutic options for chondrosarcoma.

## 2. Materials and Methods

### 2.1. Prediction of Target Genes Relevant to Drug and Disease

First of all, the related targets of Sennoside A (SA) supported by literature were obtained from several drug target databases such as STITCH, Swiss Target Prediction, and Pharmmapper, and all targets were “*Homo sapiens*.” Their websites were https://stitch.embl.de/, https://www.swisstargetprediction.ch/, and https://www.lilab-ecust.cn/pharmmapper/, respectively. Additionally, the potential targets of chondrosarcoma were predicted from databases of GeneCards and Online Mendelian Inheritance in Man (OMIM), and all targets were “*Homo sapiens*.” The websites were https://www.genecards.org/and https://www.omim.org/, respectively. Then, all targets were calibrated to uniform names through the Uniprot database, of which the website was https://www.uniprot.org/. Finally, the *R* software was used to match the predicted targets of chondrosarcoma with the possible targets of SA, taking correlation ≥1 as a threshold, so as to obtain the potential targets of SA against chondrosarcoma and draw a Venn diagram.

### 2.2. GO and KEGG Enrichment Analysis

The underlying targets of SA against chondrosarcoma were further analyzed by functional enrichment. In order to identify characteristic biological attributes and functional attributes, the gene ontology (GO) and kyoto encyclopedia of genes and genomes (KEGG) analyses were carried out, respectively. The *P* values with FDR correction less than 0.05 indicated the significant enrichment in target genes. Finally, the omicshare platform with a website of https://www.omicshare.com/tools/was used to visualize the results of GO and KEGG analyses.

### 2.3. Cell Culture and Reagents

SW1353 chondrosarcoma cell line (Cell Bank of Chinese Academy of Sciences; Shanghai, China) was cultured with Dulbeccoʼs modified Eagleʼs medium (DMEM) containing 1% double antibody and 10% fetal bovine serum (FBS) (Gibco; USA) in a cell incubator with 5% CO_2_ at 37°C. The complete DMEM was replaced every other day. SA (Sigma-Aldrich; Shanghai, China) was dissolved with dimethyl sulfoxide (Solarbio; Beijing, China). Rabbit polyclonal antibodies against Bax, Bcl-2, WNT3A, *β*-Catenin, c-Myc, and GAPDH were obtained from ABclonal Technology (Shanghai, China). Rabbit monoclonal antibodies against N-cadherin and E-cadherin were acquired from cell signaling technology (USA). Other reagents were purchased from Sigma-Aldrich (Shanghai, China) unless additional described.

### 2.4. Cell Cytotoxicity Detection

The cell counting kit-8 (CCK-8; Dojindo; Japan) was utilized to determine cell cytotoxicity. SW1353 cells (1 × 10^3^ cells/well) were inoculated into 96-well plates. The adherent cells were divided into several groups including blank, control, and SA-treated groups with a series of concentrations (5, 10, 20, 40, 80, and 100 *μ*M). After 24 h treatment, cells were incubated with a total of 100 *μ*l of DMEM containing 10% CCK-8 for 4 h. Finally, the microplate reader (Potenov; Beijing, China) was taken to detect the absorbance at 450 nm. The cell viability was calculated using the equation: Cell viability (%) = (OD_450nm_ of treatment − OD_450nm_ of blank)/(OD_450nm_ of control − OD_450nm_ of blank) × 100%. The experiment was performed thrice.

### 2.5. Colony Formation Test

SW1353 cells (1 × 10^3^ cells/well) were inoculated into 6-well plates. Then the adherent cells were divided into 4 groups including control and SA-treated groups treated with SA (40, 80 and 100 *μ*M). After 14 days incubation, cells were fixed with 4% paraformaldehyde for 15 min, and then dyed with 0.5% crystal violet for 20 min. Thereafter, the images of plates were captured and the areas of colonies were then detected by Image J software. The experiment was repeated three times.

### 2.6. Flow Cytometry Assay

SW1353 cells (1 × 10^6^ cells/well) were inoculated into 6-well plates. The adherent cells were divided into 4 groups including control and SA-treated groups treated with SA (40, 80, and 100 *μ*M). After 24 h treatment, the treated cells were washed with cold PBS buffer and stained with 5 *μ*L of Annexin V-FITC plus and 5 *μ*L of propidium iodide (Dojindo; Japan) at 4°C for 30 minutes in dark. The stained cells were washed by binding buffer 3 times to remove excess dyes and then resuspended in 500 *μ*L of binding buffer. Finally, the flow cytometer (BD Biosciences; USA) was taken to analyze the percentage of apoptosis, and FlowJo 10.4 software was utilized for quantitative analysis. The experiment was performed in triplicate.

### 2.7. Wound Healing Scratch Test

Ibidi culture inserts (Ibidi; Germany) were set onto 6-well plates. Then a total of 70 *μ*l of cell suspension with 7 × 10^4^ SW1353 cells were inoculated into the well of Ibidi isolation chambers. After the cells had adhered to the wall and grown to 100% confluence, the Ibidi insert was gently removed, leaving an approximately 500 *μ*m wide gap. Subsequently, the control and SA-treated groups were treated by 0, 40, 80, and 100 *μ*M SA for 24 h. Finally, a 100× inverted microscope (Leica Microsystems; Germany) was used to take images of plates after 0, 12, and 24 h treatment, and the rate of wound closure was measured by Image J software. The experiment was repeated three times.

### 2.8. Transwell Assays

The transwell chambers (Corning; USA) were coated with and without matrigel to detect the ability of invasion and migration in SW1353 cells, respectively. After the treatment of 0, 40, 80, and 100 *μ*M SA for 24 h, a total of 1 × 10^5^ SW1353 cells were incubated with 200 *μ*l of low serum DMEM with 1% FBS and seeded onto the upper transwell chamber. Besides, 600 *μ*l of complete DMEM with 10% FBS was added into the lower chamber. After 24 h incubation, the migrated and invaded cells on the reverse side of the upper transwell chamber were fixed with 4% paraformaldehyde and then stained with 0.5% crystal violet. Finally, the 100× inverted microscope was used to take pictures of these stained cells in 5 random fields. The experiment was performed thrice.

### 2.9. Western Blot Assay

SW1353 cells (1 × 10^6^ cells/well) were inoculated into 6-well plates. The adherent cells were divided into 2 groups including control and 100 *μ*M SA-treated groups. The proteins were extracted from cell lysates in the ice-cold radioimmunoprecipitation buffer (EpiZyme; Shanghai, China) after 24 h treatment and quantified using the BCA Protein Assay Kit (YEASEN; Shanghai, China). A total of 20 *μ*g proteins were separated by SDS-PAGE and then transferred onto the PVDF membranes (Millipore; USA). Following blocking by 5% BSA buffer, the membranes were incubated with primary antibodies against Bax, Bcl-2, N-cadherin, E-cadherin, WNT3A, *β*-catenin, c-Myc, and GAPDH (diluted 1 : 1000) for overnight at 4°C and further secondary antibodies (diluted 1 : 10000; ABclonal; Shanghai, China) at RT for 2 h. Finally, the ECL detection reagent (EpiZyme; Shanghai, China) was used for the visualization of the proteins. The experiment was performed in triplicate.

### 2.10. Statistical Analysis

All measured data were presented as means ± SEM. Differences were assessed by using Student's *t*-test with 95% confidence level in two independent samples. Comparisons for more than two groups were evaluated using one-way ANOVA followed by POST HOC LSD. All statistical analyses were performed using SPSS 23.0 software. The statistical significance was defined as *P* < 0.05.

## 3. Results

### 3.1. The Potential Targets Associated with SA against Chondrosarcoma

**Figure 1:**
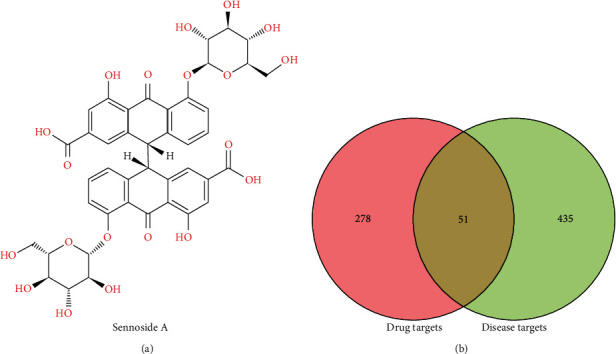
The potential targets associated with SA against chondrosarcoma. (a) SA is a natural compound with the chemical structure of dianthrone glycosides. (b) The possible target genes associated with SA against chondrosarcoma were obtained by intersection of drug- and disease-related targets.

According to the chemical structure of SA (Figure 1(a)), a total of 329 possible SA-related targets were predicted using STITCH, Swiss Target Prediction, and Pharmmapper, and annotated through the Uniprot database (Supplement Table 1). Besides, a total of 811 chondrosarcoma-related targets were acquired from the GeneCards and OMIM databases, of which 486 genes with relevance score more than 1 were retained (Supplement Table 2). Ultimately, a total of 51 underlying targets of SA against chondrosarcoma were screened through the intersection of the above two targets, which were used for further research (Figure 1(b) and Supplement Table 3).

### 3.2. GO Analysis of Potential Targets

**Figure 2:**
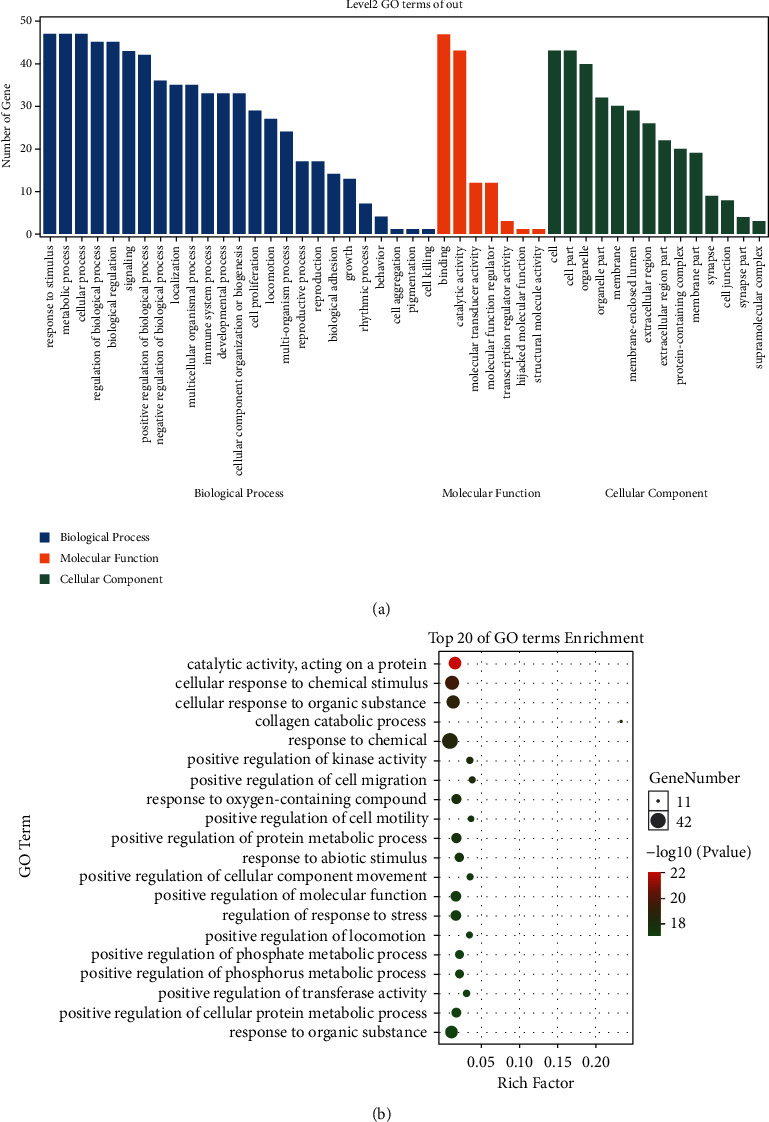
GO analysis of potential targets. (a) The enrichment GO terms were annotated and classified into 3 categories including biological process (BP), molecular function (MF), and cellular component (CC). (b) The top 20 GO functional enrichment analysis.

As shown in GO enrichment analysis (Figure 2(a)), the most significant functions of the 51 underlying targets of SA in biological process (BP) were the cellular process, metabolic process, and response to stimulus; the main representative subcategories in molecular function (MF) contained the binding, catalytic activity, and molecular transducer activity; the terms of the highest percentages in cellular component (CC) included the cell, organelle, and cell part. The top 20 of GO terms were enriched in the catalytic activity acting on a protein, response to chemical/organic/abiotic stimulus, positive regulation of cell migration/cell motility/cellular component movement//locomotion/molecular function/cellular protein metabolic process/transferase activity/kinase activity, which were also connected with the initiation and development of tumors (Figure 2(b)).

### 3.3. KEGG Analysis of Potential Targets

**Figure 3:**
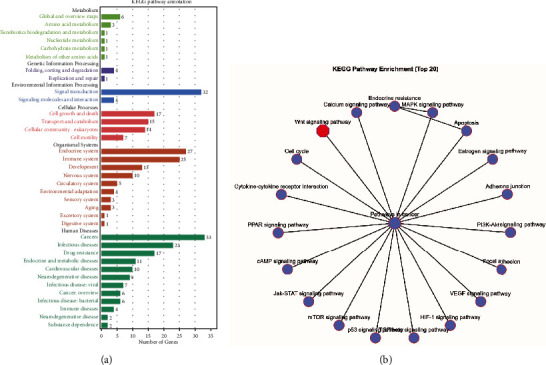
KEGG analysis of potential targets. (a) The enrichment KEGG pathways were annotated and divided into 6 categories and 36 subcategories, among which human diseases and cancers were the uppermost categories and subcategories, respectively. (b) The top 20 KEGG enrichment advanced network diagram. The red mark represents the WNT pathway.

Next, we further defined the biological functions and signaling pathways of 51 target genes of SA against chondrosarcoma using the KEGG enrichment analysis. The KEGG pathway enrichment analysis clustered the 51 target genes into 6 major categories and further subdivided into 36 subcategories, of which human diseases and cancers were the most major category and subcategory (Figure 3(a)). The top 20 of KEGG enrichment pathways contained pathways in cancer related to cell cycle, focal adhesion, adherens junction, and apoptosis, such as Wnt, PPAR, cAMP, mTOR, Jak-STAT, p53, TGF-beta, HIF-1, VEGF, MAPK, and PI3K-Akt signaling pathways, which all participated in tumorigenesis and metastasis (Figure 3(b)).

### 3.4. SA Stimulated the Cytotoxicity and Suppressed the Proliferation in Chondrosarcoma Cells

**Figure 4:**
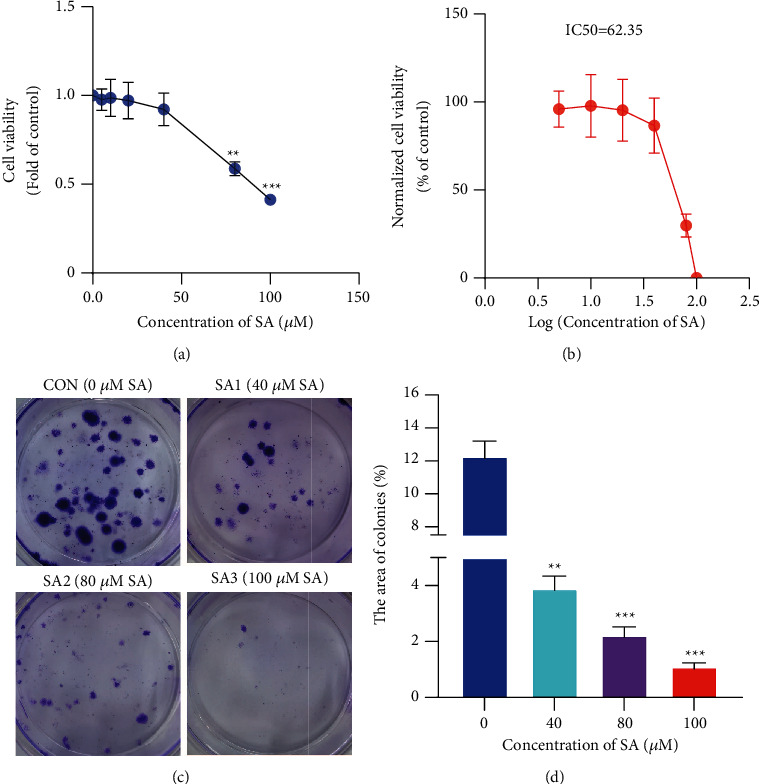
SA stimulated the cytotoxicity and suppressed the proliferation in chondrosarcoma cells. (a) The cell cytotoxicity and (b) the IC50 values were detected through CCK-8 method. (c) The colonies were dyed with crystal violet. (d) The area of colonies of SW1353 cells was counted using Image J software. Data are expressed as mean ± SEM (*n* = 3). *∗∗∗p* < 0.001, *∗∗p* < 0.01, *∗p* < 0.05 vs. control.

The results of CCK-8 cytotoxicity assay confirmed that SA effectively induced the cytotoxicity of SW1353 chondrosarcoma cells with an IC50 at 62.35 *μ*M (Figures 4(a) and 4(b)). Consistent with the CCK-8 results, the area of colonies of SW1353 cells was decreased obviously from (12.27 ± 0.94)% in the control group to (3.89 ± 0.44)%, (2.22 ± 0.30)% and (1.09 ± 0.15)% in the treatment groups with 40, 80, and 100 *μ*M of SA, respectively (Figures 4(c) and 4(d)). These data clearly demonstrated that SA stimulated the cytotoxicity and inhibited the proliferation of SW1353 cells in a dose-dependent manner.

### 3.5. SA Induced the Apoptosis in Chondrosarcoma Cells

**Figure 5:**
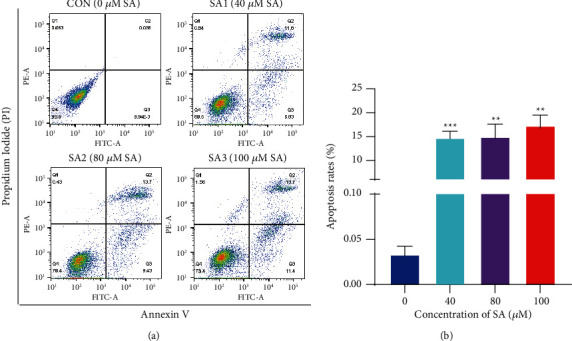
SA induced the apoptosis in chondrosarcoma cell. (a) The apoptotic cells were found in the regions of Annexin V-FITC ^+^/PI^−^ and Annexin V-FITC ^+^/PI^+^ (PI, propidium iodide; FITC, fluorescein isothiocyanate). (b) The apoptosis rates were quantified by FlowJo software. Data are expressed as mean ± SEM (*n* = 3). ^*∗∗∗*^*p* < 0.001, ^*∗∗*^*p* < 0.01, ^*∗*^*p* < 0.05 vs. control.

Then, we analyzed whether the inhibition of SA in chondrosarcoma cell growth is related to apoptosis. The flow cytometry assay of PI and Annexin V was used to determine SA-induced apoptosis in SW1353 cells (Figure 5(a)). The apoptosis rate was enhanced from (0.03 ± 0.01)% in the control group to (14.7 ± 1.42)%, (14.95 ± 2.68)%, and (17.25 ± 2.26)% upon 24 h treatment with 40, 80, and 100 *μ*M SA, respectively (Figure 5(b)). In a word, the results demonstrated that SA significantly and dose-dependently induced the apoptosis of SW1353 chondrosarcoma cells.

### 3.6. SA Inhibited the Metastasis in Chondrosarcoma Cells

**Figure 6:**
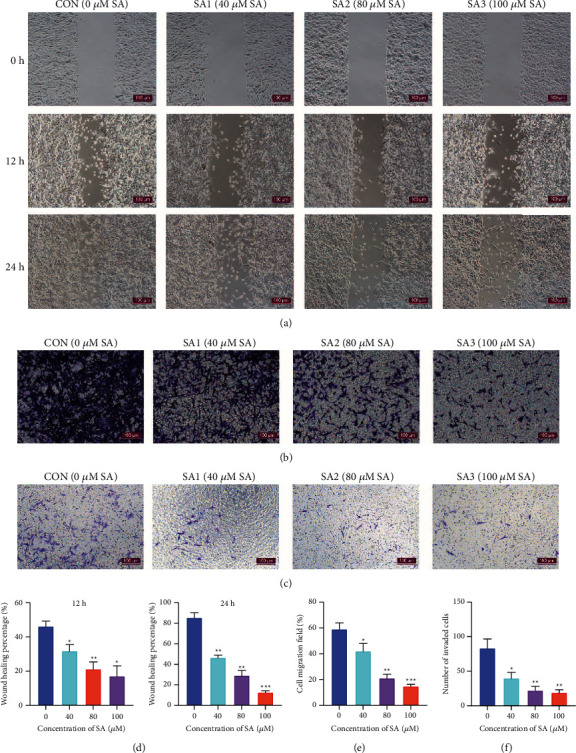
SA inhibited the metastasis of chondrosarcoma cells. (a) The scratch assay was utilized to test the migration of SW1353 cells treated by SA for 12h and 24 h. (b) The migration and (c) the invasion of SW1353 cells treated by SA were measured by the transwell experiments. (d) The percentage of wound healing, (e) the field of cell migration, and (f) the number of invaded cells were counted by Image J software. Data are expressed as mean ± SEM (*n* = 3). ^*∗∗∗*^*p* < 0.001, ^*∗∗*^*p* < 0.01, ^*∗*^*p* < 0.05 vs. control.

Firstly, we used the wound healing experiments to test the effect of SA on the migration of SWA353 cells. After 12 h of treatment with 40, 80, and 100 *μ*M SA, the percentage of wound healing was reduced from (46.20 ± 3.09)% in the control group to (31.76 ± 3.79)%, (21.19 ± 4.16)%, and (17.15 ± 6.00)%, respectively (Figures 6(a) and 6(d)). Subsequently, the percentage was decreased from (85.50 ± 4.67)% in the control group to (46.50 ± 2.41)%, (29.12 ± 4.85)%, and (12.62 ± 1.46)%, respectively after 24 h treatment with 40, 80, and 100 *μ*M SA (Figures 6(a) and 6(d)).

Meanwhile, we detected the SW1353 cell invasion by using transwell experiments. Transwell migration assays showed that the percentage of migration field decreased significantly from (59.09 ± 4.77)% in the control group to (42.03 ± 6.02)%, (21.06 ± 3.03)%, and (14.80 ± 1.51)% upon treatment with 40, 80, and 100 *μ*M SA, respectively (Figures 6(b) and 6(e)). The number of invaded cells was suppressed from 83.50 ± 13.23 in the control group to 39.80 ± 8.52, 22.30 ± 5.72, and 19.20 ± 3.99 upon treatment with 40, 80, and 100 *μ*M SA, respectively (Figures 6(c) and 6(f)). The above results verified that SA remarkably restrained the metastasis of chondrosarcoma cells in a dose-dependent manner.

### 3.7. SA Negatively Regulated Apoptosis, Metastasis, and the Wnt/*β*-Catenin Pathway in Chondrosarcoma Cells

**Figure 7:**
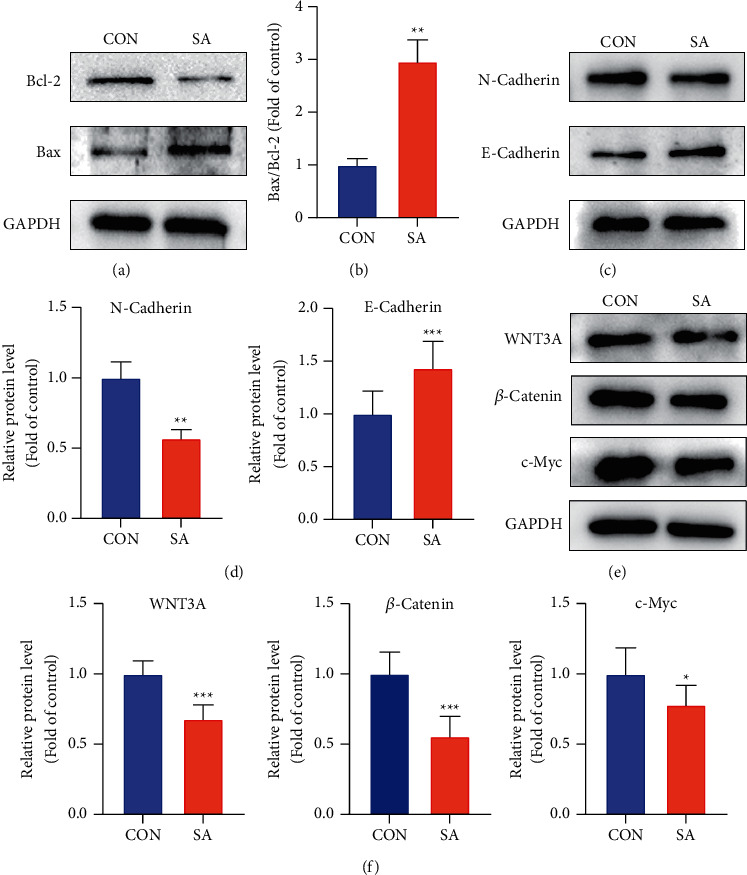
SA negatively regulated apoptosis, metastasis, and the Wnt/*β*-catenin pathways in chondrosarcoma cells. The levels of proteins relevant to apoptosis, metastasis, and the Wnt/*β*-catenin pathways were, respectively, quantified by western blotting. (a, b) The apoptosis-related proteins (Bcl-2, Bax) in SW1353 cells. (c, d) The metastasis-related proteins (E-cadherin, N-cadherin) in SW1353 cells. (e, f) Proteins in the Wnt/*β*-catenin pathway (WNT3A, *β*-Catenin, and c-Myc) in SW1353 cells. Relative protein expression was assayed by normalizing with GAPDH. Data are expressed as mean ± SEM (*n* = 3). ^*∗∗∗*^*p* < 0.001, ^*∗∗*^*p* < 0.01, ^*∗*^*p* < 0.05 vs. control.

The pro-apoptosis mechanism of SA was further investigated. In line with the results of cell apoptosis assay, SA apparently upregulated the proapoptotic protein Bax, downregulated the antiapoptotic protein Bcl-2, and resulted in an increase in the protein ratio of Bax/Bcl-2 (Figures 7(a) and 7(b)). Furthermore, the effects of SA on the levels of metastasis-related proteins were investigated. Consistent with the results of cell migration and invasion experiments, SA significantly downregulated the expression of tumor metastasis marker N-cadherin but upregulated E-cadherin levels (Figures 7(c) and 7(d)).

It is well known that the Wnt/*β*-catenin pathway plays a central role in the proliferation, apoptosis, and metastasis of cancer cells. To determine whether the Wnt/*β*-catenin pathway participated in the anticancer effect of SA on chondrosarcoma cells, we examined the related protein expression of the Wnt/*β*-catenin pathway in SW1353 chondrosarcoma cells. The results illustrated that SA obviously decreased the protein levels of Wnt3a, *β*-catenin, and c-Myc in SW1353 cells (Figures 7(e) and 7(f)). Overall, the above results indicated that SA might induce cell apoptosis and restrain cell growth and metastasis of chondrosarcoma cells via inhibiting the Wnt/*β*-catenin pathway.

## 4. Discussion

There are few studies on the anticancer effect of SA, and only the possible inhibitory effects of SA on pancreatic cancer and HCC have been reported. Sennoside B (SB), the diastereomer of SA, has been reported to suppress downstream pathway of PDGFR-*β* by binding to extracellular domains of PDGF-BB and its receptor. Due to its binding properties, SB can inhibit the proliferative effect of PDGF in MG63 human osteoblast-like cells, while SA does not have this binding effect and antiproliferation effect possibly due to conformational changes [11]. At present, the role and molecular mechanism of SA in chondrosarcoma cells remains really unclear. Our study aimed to confirm the antitumor activity of SA against chondrosarcoma cells and reveal the underlying mechanisms using both network pharmacology method and validation experiments in vitro. The results indicated that SA suppressed proliferation and metastasis, and facilitated cytotoxicity and apoptosis in SW1353 chondrosarcoma cells, possibly by inhibiting the Wnt/*β*-catenin pathway.

The classical Wnt signaling pathway, namely, the Wnt/*β*-catenin pathway, is an extremely complex and unique pathway. It plays a critical role in regulating tumor biological characteristics and behaviors, such as tumor metastasis, proliferation, differentiation, apoptosis, and tumor gene expression [12]. As a subunit of the cadherin complex, *β*-catenin has a key effect on the transduction of downstream signals in the Wnt signaling pathway. It regulates the content of key genes connected with the developmental processes of cancer and therefore plays a central role in carcinogenesis [13]. Wnt ligands enable the Wnt/*β*-catenin pathway by binding to Frizzled 7-pass transmembrane receptors and low-density lipoprotein-related receptors 5/6. Subsequently, *β*-catenin accumulates in the cytoplasm and is transported into the nucleus, where it binds with TCF/LEF transcription factors to mediate transcriptional induction of downstream oncogenes [14, 15]. However, in the inactivated state of the Wnt/*β*-catenin pathway, *β*-catenin cannot accumulate in the cytoplasm for subsequent effects due to its degradation by the destruction complex, which includes a variety of proteins, such as Axin, GSK-3*β*, and adenomatous polyposis coli [16–18].

In this study, we found a decrease of the protein levels in the Wnt/*β*-catenin pathway, including Wnt3a, *β*-catenin, and its downstream effector c-Myc in SA-treated SW1353 cells, which suggested that SA might exert its anticancer effects by deactivating the Wnt/*β*-catenin pathway. The Wnt/*β*-catenin pathway has been considered to participate in promoting the progress of epithelial mesenchymal transition (EMT) and cell metastasis in several types of cancer [19–21]. EMT is a developmental program with conserved evolution involved in tumorigenesis that improves mobility, invasiveness, and resistance to apoptotic stimulation of cancer cells, thereby conferring them with metastatic characteristics [22, 23]. The tumor cells undergoing EMT typically exhibit some changes in molecular biology, such as an upregulation of mesenchymal markers such as N-cadherin but a downregulation of epithelial markers such as E-cadherin [24, 25]. Consistently, our data have certified that SA downregulated N-cadherin expression but upregulated E-cadherin level in SW1353 cells, which suggested that SA inhibited EMT and metastasis of chondrosarcoma cells probably by inactivating the Wnt/*β*-catenin pathway.

Furthermore, apoptosis, a major type of programmed cell death, plays an important role in the development and progression of tumors [26]. The Bcl-2 family contains a series of key apoptotic regulators participating in the apoptotic process of cancer, which are abnormally expressed in many types of cancer cells [27]. The proteins of Bcl-2 family are composed of both antiapoptotic factors such as Bcl-W, Bcl-XL, Bcl-2, and proapoptotic factors such as Bad, Bak, Bax [28]. The Bax/Bcl-2 ratio determines cell susceptibility to apoptosis and high Bax/Bcl-2 ratio indicates the enhanced apoptosis of cancer cells [29]. Analogously, SA markedly induced apoptosis in SW1353 cells, which was evidenced by increased apoptotic cells and higher Bax/Bcl-2 ratio. The results of this experiment confirmed that the anticancer mechanism of SA was related to intracellular mitochondrial apoptosis of cancer cells.

## 5. Conclusion

**Figure 8:**
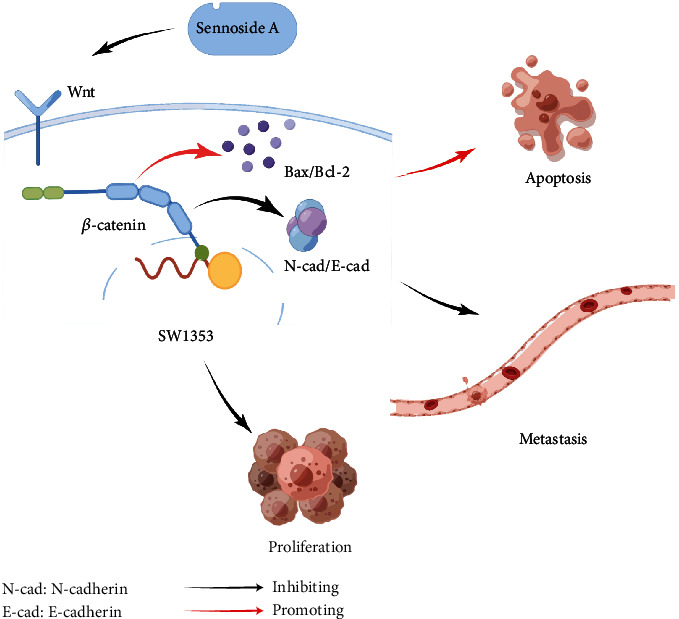
The underlying mechanism of Sennoside A for induction of cytotoxicity and apoptosis, inhibition of proliferation and metastasis in human chondrosarcoma cells.

Taken together, SA could effectively stimulate cytotoxicity to restrain the growth of cell, suppress the metastasis of cell by inhibiting EMT, and promote the death of cell via inducing apoptosis in SW1353 chondrosarcoma cells. The mechanism of anticancer activity might be concerned with the SA-mediated inhibition of the Wnt/*β*-catenin pathway in chondrosarcoma cells (Figure 8). Therefore, this study verified that SA might be a promising agent against human chondrosarcoma cells in vitro experiment. However, the main research limitation is the lack of validation in more physiological models and in vivo experiments. The specific molecular targets and mechanisms of SA in regulating tumor metastasis and the WNT/*β*-catenin pathway, as well as its participation in the destruction complex of *β*-catenin also remain unclear and need further study.

## Data Availability

The data used to support the findings of this study are available from the corresponding author upon request.

## Supplementary Materials

Supplementary Materials Supplement Table 1: the predicted targets of Sennoside A (SA). Supplement Table 2: the possible targets of chondrosarcoma. Supplement Table 3: the potential targets associated with SA against chondrosarcoma.

## References

[1] MacDonald I. J., Lin C. Y., Kuo S. J., Su C. M., Tang C. H. (2019). An update on current and future treatment options for chondrosarcoma. *Expert Review of Anticancer Therapy*.

[2] Miwa S., Yamamoto N., Hayashi K., Takeuchi A., Igarashi K., Tsuchiya H. (2022). Therapeutic targets and emerging treatments in advanced chondrosarcoma. *International Journal of Molecular Sciences*.

[3] Italiano A., Mir O., Cioffi A. (2013). Advanced chondrosarcomas: role of chemotherapy and survival. *Annals of Oncology: Official Journal of the European Society for Medical Oncology*.

[4] Girard N., Lhuissier E., Aury-Landas J. (2020). Heterogeneity of chondrosarcomas response to irradiations with X-rays and carbon ions: a comparative study on five cell lines. *Journal of Bone Oncology*.

[5] Jin H., Chen H., Yu K. (2016). Resveratrol inhibits phosphorylation within the signal transduction and activator of transcription 3 signaling pathway by activating sirtuin 1 in SW1353 chondrosarcoma cells. *Molecular Medicine Reports*.

[6] Zhu M., Ying J., Lin C. (2019). Baicalin induces apoptotic death of human chondrosarcoma cells through mitochondrial dysfunction and downregulation of the PI3K/Akt/mTOR pathway. *Planta Medica*.

[7] Zhang H.-T., Yang J., Liang G.-H. (2017). Andrographolide induces cell cycle arrest and apoptosis of chondrosarcoma by targeting TCF-1/SOX9 Axis. *Journal of Cellular Biochemistry*.

[8] Le J., Ji H., Zhou X. (2021). Pharmacology, toxicology, and metabolism of sennoside A, A medicinal plant-derived natural compound. *Frontiers in Pharmacology*.

[9] Lee S. Y., Kim W., Lee Y. G. (2017). Identification of sennoside A as a novel inhibitor of the slingshot (SSH) family proteins related to cancer metastasis. *Pharmacological Research*.

[10] Le J., Fu Y., Han Q. (2021). Transcriptome analysis of the inhibitory effect of sennoside A on the metastasis of hepatocellular carcinoma cells. *Frontiers Pharmacology*.

[11] Chen Y.-C., Chang C.-N., Hsu H.-C., Chiou S.-J., Lee L.-T., Hseu T.-H. (2009). Sennoside B inhibits PDGF receptor signaling and cell proliferation induced by PDGF-BB in human osteosarcoma cells. *Life Sciences*.

[12] Nusse R., Clevers H. (2017). Wnt/*β*-catenin signaling, disease, and emerging therapeutic modalities. *Cell*.

[13] Zhang X., Wang L., Qu Y. (2020). Targeting the *β*-catenin signaling for cancer therapy. *Pharmacological Research*.

[14] Zhang Y., Wang X. (2020). Targeting the Wnt/*β*-catenin signaling pathway in cancer. *Journal of Hematology & Oncology*.

[15] Tian J., He H., Lei G. (2014). Wnt/*β*-catenin pathway in bone cancers. *Tumour Biology: The Journal of the International Society for Oncodevelopmental Biology and Medicine*.

[16] Mao J., Wang J., Liu B. (2001). Low-density lipoprotein receptor-related protein-5 binds to Axin and regulates the canonical Wnt signaling pathway. *Molecular Cell*.

[17] Zeng X., Huang H., Tamai K. (2008). Initiation of Wnt signaling: control of Wnt coreceptor Lrp6 phosphorylation/activation via frizzled, dishevelled and axin functions. *Development*.

[18] He X., Semenov M., Tamai K., Zeng X. (2004). LDL receptor-related proteins 5 and 6 in Wnt/beta-catenin signaling: arrows point the way. *Development*.

[19] Gonzalez D. M., Medici D. (2014). Signaling mechanisms of the epithelial-mesenchymal transition. *Science Signaling*.

[20] Clevers H. (2004). Wnt breakers in colon cancer. *Cancer Cell*.

[21] Said N. A. B. M., Williams E. D. (2011). Growth factors in induction of epithelial-mesenchymal transition and metastasis. *Cells, Tissues, Organs*.

[22] Mittal V. (2018). Epithelial mesenchymal transition in tumor metastasis. *Annual Review of Pathology*.

[23] Rhim A. D., Mirek E. T., Aiello N. M. (2012). EMT and dissemination precede pancreatic tumor formation. *Cell*.

[24] Christofori G., Semb H. (1999). The role of the cell-adhesion molecule E-cadherin as a tumour-suppressor gene. *Trends in Biochemical Sciences*.

[25] Nieman M. T., Prudoff R. S., Johnson K. R., Wheelock M. J. (1999). N-cadherin promotes motility in human breast cancer cells regardless of their E-cadherin expression. *The Journal of Cell Biology*.

[26] Ouyang L., Shi Z., Zhao S. (2012). Programmed cell death pathways in cancer: a review of apoptosis, autophagy and programmed necrosis. *Cell Proliferation*.

[27] Llambi F., Green D. R. (2011). Apoptosis and oncogenesis: give and take in the BCL-2 family. *Current Opinion in Genetics & Development*.

[28] Deng J. (2017). How to unleash mitochondrial apoptotic blockades to kill cancers. *Acta Pharmaceutica Sinica B*.

[29] Li X., Ye H., Cai L. (2012). Millimeter wave radiation induces apoptosis via affecting the ratio of Bax/Bcl-2 in SW1353 human chondrosarcoma cells. *Oncology Report*.

