# Supplementary material for: The Effects and Mechanisms of Sennoside A on Inducing Cytotoxicity, Apoptosis, and Inhibiting Metastasis in Human Chondrosarcoma Cells
Source: Evid Based Complement Alternat Med. 2022 Aug 31;2022:8063497. doi: 10.1155/2022/8063497 (PMC9451980; doi:10.1155/2022/8063497)
Supplement: Supplementary Materials — Supplement Table 1: the predicted targets of Sennoside A (SA). Supplement Table 2: the possible targets of chondrosarcoma. Supplement Table 3: the potential targets associated with SA against chondrosarcoma. [file 8063497.f1.pdf]

Supplementary Materials

Supplement Table 1. The predicted targets of Sennoside A (SA).

SYMBOL	ENTREZID	ENSEMBL	REFSEQ
CA12	771	ENSG00000074410	NM_001218
EEA1	8411	ENSG00000102189	NM_003566
CASP3	836	ENSG00000164305	NM_001354777
BMP2	650	ENSG00000125845	NM_001200
TTR	7276	ENSG00000118271	NM_000371
CDK2	1017	ENSG00000123374	NM_001290230
GSTP1	2950	ENSG00000084207	NM_000852
MAPK10	5602	ENSG00000109339	NM_001318067
CMA1	1215	ENSG00000092009	NM_001308083
PDE4B	5142	ENSG00000184588	NM_001037339
FAP	2191	ENSG00000078098	NM_001291807
PPIA	5478	ENSG00000196262	NM_001300981
GBA	2629	ENSG00000177628	NM_000157
GBA	2629	ENSG00000262446	NM_000157
HSD17B11	51170	ENSG00000198189	NM_016245
PTPN1	5770	ENSG00000196396	NM_001278618
CTSD	1509	ENSG00000117984	NM_001909
BCHE	590	ENSG00000114200	NM_000055
PNP	4860	ENSG00000198805	NM_000270
KDR	3791	ENSG00000128052	NM_002253
ICAM2	3384	ENSG00000108622	NM_000873
TGFBR2	7048	ENSG00000163513	NM_001024847
RTN4R	65078	ENSG00000040608	NM_023004
FCAR	2204	ENSG00000186431	NM_002000
FCAR	2204	ENSG00000275269	NM_002000
FCAR	2204	ENSG00000273738	NM_002000
FCAR	2204	ENSG00000276985	NM_002000
FCAR	2204	ENSG00000276858	NM_002000
FCAR	2204	ENSG00000275136	NM_002000
FCAR	2204	ENSG00000275970	NM_002000
FCAR	2204	ENSG00000278415	NM_002000
FCAR	2204	ENSG00000275564	NM_002000
FCAR	2204	ENSG00000274580	NM_002000
FCAR	2204	ENSG00000283750	NM_002000
FCAR	2204	ENSG00000283953	NM_002000
FCAR	2204	ENSG00000284061	NM_002000
FCAR	2204	ENSG00000284004	NM_002000
FCAR	2204	ENSG00000284245	NM_002000

PIM1	5292	ENSG00000137193	NM_001243186
AKR1B1	231	ENSG00000085662	NM_001346142
FKBP1A	2280	ENSG00000088832	NM_000801
PGR	5241	ENSG00000082175	NM_000926
METAP1	23173	ENSG00000164024	NM_015143
GSR	2936	ENSG00000104687	NM_000637
CHIT1	1118	ENSG00000133063	NM_001256125
CLPP	8192	ENSG00000125656	NM_006012
HSPA8	3312	ENSG00000109971	NM_006597
LGALS7	3963	ENSG00000205076	NM_002307
LGALS7	3963	ENSG00000282902	NM_002307
LGALS7	3963	ENSG00000283082	NM_002307
LGALS7	3963	ENSG00000178934	NM_002307
CA2	760	ENSG00000104267	NM_000067
PKLR	5313	ENSG00000143627	NM_000298
PKLR	5313	ENSG00000262785	NM_000298
ALB	213	ENSG00000163631	NM_000477
APOA2	336	ENSG00000158874	NM_001643
AKR1C2	1646	ENSG00000151632	NM_001135241
BACE1	23621	ENSG00000186318	NM_001207048
CTSV	1515	ENSG00000136943	NM_001201575
EPHB4	2050	ENSG00000196411	NM_004444
CA1	759	ENSG00000133742	NM_001128829
ESR1	2099	ENSG00000091831	NM_000125
CASP7	840	ENSG00000165806	NM_001227
NR1H2	7376	ENSG00000131408	NM_001256647
HCK	3055	ENSG00000101336	NM_001172129
IMPA1	3612	ENSG00000133731	NM_001144878
TREM1	54210	ENSG00000124731	NM_001242589
CSNK2A1	1457	ENSG00000101266	NM_001362770
RNASE4	6038	ENSG00000258818	NM_001282192
THRB	7068	ENSG00000151090	NM_000461
PLAU	5328	ENSG00000122861	NM_001145031
IGF1R	3480	ENSG00000140443	NM_000875
LDHB	3945	ENSG00000111716	NM_001174097
SRC	6714	ENSG00000197122	NM_005417
MAPK1	5594	ENSG00000100030	NM_002745
PSPH	5723	ENSG00000146733	NM_001370503
ANG	283	ENSG00000214274	NM_001097577
DHFR	1719	ENSG00000228716	NM_000791
CPB1	1360	ENSG00000153002	NM_001871
CCNA2	890	ENSG00000145386	NM_001237
ME2	4200	ENSG00000082212	NM_001168335
SHBG	6462	ENSG00000129214	NM_001040

MMP8	4317	ENSG00000118113	NM_001304441
CHEK1	1111	ENSG00000149554	NM_001114121
SULT2A1	6822	ENSG00000105398	NM_003167
F2	2147	ENSG00000180210	NM_000506
PNPO	55163	ENSG00000108439	NM_018129
MMP3	4314	ENSG00000149968	NM_002422
GC	2638	ENSG00000145321	NM_000583
BMP7	655	ENSG00000101144	NM_001719
MTAP	4507	ENSG00000099810	NM_002451
APCS	325	ENSG00000132703	NM_001639
CRAT	1384	ENSG00000095321	NM_000755
MMP13	4322	ENSG00000137745	NM_002427
CFD	1675	ENSG00000197766	NM_001317335
CFD	1675	ENSG00000274619	NM_001317335
EGFR	1956	ENSG00000146648	NM_001346897
MAPK14	1432	ENSG00000112062	NM_001315
CTSB	1508	ENSG00000164733	NM_001317237
CTSB	1508	ENSG00000285132	NM_001317237
AMY2A	279	ENSG00000243480	NM_000699
BCAT2	587	ENSG00000105552	NM_001164773
IGLV2-8	28817	ENSG00000278196	NA
FABP4	2167	ENSG00000170323	NM_001442
AZGP1	563	ENSG00000160862	NM_001185
HSD17B1	3292	ENSG00000108786	NM_000413
ADH1B	125	ENSG00000196616	NM_000668
F10	2159	ENSG00000126218	NM_000504
NOS3	4846	ENSG00000164867	NM_000603
MAOB	4129	ENSG00000069535	NM_000898
PDE4D	5144	ENSG00000113448	NM_001104631
GALK1	2584	ENSG00000108479	NM_000154
PPP5C	5536	ENSG00000011485	NM_001204284
CFB	629	ENSG00000243649	NM_001710
CFB	629	ENSG00000242335	NM_001710
CFB	629	ENSG00000204359	NM_001710
CFB	629	ENSG00000243570	NM_001710
CFB	629	ENSG00000241534	NM_001710
CFB	629	ENSG00000239754	NM_001710
CFB	629	ENSG00000241253	NM_001710
CSNK1G2	1455	ENSG00000133275	NM_001319
PYGL	5836	ENSG00000100504	NM_001163940
SORD	6652	ENSG00000140263	NM_003104
NMNAT3	349565	ENSG00000163864	NM_001200047
AHCY	191	ENSG00000101444	NM_000687
HSP90AA1	3320	ENSG00000080824	NM_001017963

REN	5972	ENSG00000143839	NM_000537
HSD11B1	3290	ENSG00000117594	NM_001206741
AKR1C3	8644	ENSG00000196139	NM_001253908
KIF11	3832	ENSG00000138160	NM_004523
KIF5B	3799	ENSG00000170759	NM_004521
PAH	5053	ENSG00000171759	NM_000277
PDE3B	5140	ENSG00000152270	NM_000922
NCS1	23413	ENSG00000107130	NM_001128826
PDPK1	5170	ENSG00000140992	NM_001261816
OTC	5009	ENSG00000036473	NM_000531
DHODH	1723	ENSG00000102967	NM_001025193
ARF1	375	ENSG00000143761	NM_001024226
C1S	716	ENSG00000182326	NM_001346850
TGFBR1	7046	ENSG00000106799	NM_001130916
NR3C2	4306	ENSG00000151623	NM_000901
IMPDH2	3615	ENSG00000178035	NM_000884
AKT1	207	ENSG00000142208	NM_001014431
MMP12	4321	ENSG00000262406	NM_002426
GALE	2582	ENSG00000117308	NM_000403
FGF1	2246	ENSG00000113578	NM_000800
TGM3	7053	ENSG00000125780	NM_003245
EPHA2	1969	ENSG00000142627	NM_001329090
PARP1	142	ENSG00000143799	NM_001618
TPI1	7167	ENSG00000111669	NM_000365
ANXA5	308	ENSG00000164111	NM_001154
PLA2G2A	5320	ENSG00000188257	NM_000300
TYMS	7298	ENSG00000176890	NM_001071
PCK1	5105	ENSG00000124253	NM_002591
DPP4	1803	ENSG00000197635	NM_001379604
MAPKAPK2	9261	ENSG00000162889	NM_004759
TGM2	7052	ENSG00000198959	NM_001323316
CLIC1	1192	ENSG00000213719	NM_001287593
CLIC1	1192	ENSG00000223639	NM_001287593
CLIC1	1192	ENSG00000226417	NM_001287593
CLIC1	1192	ENSG00000226651	NM_001287593
CLIC1	1192	ENSG00000226248	NM_001287593
CLIC1	1192	ENSG00000230685	NM_001287593
CLIC1	1192	ENSG00000206394	NM_001287593
PDE5A	8654	ENSG00000138735	NM_001083
AR	367	ENSG00000169083	NM_000044
RNASE3	6037	ENSG00000169397	NM_002935
PLK1	5347	ENSG00000166851	NM_005030
HMGCR	3156	ENSG00000113161	NM_000859
LGALS2	3957	ENSG00000100079	NM_006498

UCK2	7371	ENSG00000143179	NM_001363568
ABO	28	ENSG00000175164	NM_020469
ABO	28	ENSG00000281879	NM_020469
SOD2	6648	ENSG00000112096	NM_000636
CTSK	1513	ENSG00000143387	NM_000396
RXRA	6256	ENSG00000186350	NM_001291920
PDHB	5162	ENSG00000168291	NM_000925
RHEB	6009	ENSG00000106615	NM_005614
FHIT	2272	ENSG00000189283	NM_001166243
LCK	3932	ENSG00000182866	NM_001042771
GSK3B	2932	ENSG00000082701	NM_001146156
BAG1	573	ENSG00000107262	NM_001172415
B3GAT1	27087	ENSG00000109956	NM_001367973
PPARG	5468	ENSG00000132170	NM_001330615
ADH1C	126	ENSG00000248144	NM_000669
MIF	4282	ENSG00000240972	NM_002415
MIF	4282	ENSG00000276701	NM_002415
INSR	3643	ENSG00000171105	NM_000208
MTHFD1	4522	ENSG00000100714	NM_001364837
DAPK1	1612	ENSG00000196730	NM_001288729
ISG20	3669	ENSG00000172183	NM_001303233
TYMP	1890	ENSG00000025708	NM_001113755
AKR1C1	1645	ENSG00000187134	NM_001353
F7	2155	ENSG00000057593	NM_000131
MAPK8	5599	ENSG00000107643	NM_001278547
BST1	683	ENSG00000109743	NM_004334
SERPINA1	5265	ENSG00000197249	NM_000295
SERPINA1	5265	ENSG00000277377	NM_000295
MMP7	4316	ENSG00000137673	NM_002423
ACP3	55	ENSG00000014257	NM_001099
RAC2	5880	ENSG00000128340	NM_002872
ITK	3702	ENSG00000113263	NM_005546
YARS1	8565	ENSG00000134684	NM_003680
DCK	1633	ENSG00000156136	NM_000788
CDA	978	ENSG00000158825	NM_001785
C1R	715	ENSG00000159403	NM_001354346
C1R	715	ENSG00000288512	NM_001354346
XIAP	331	ENSG00000101966	NM_001167
FNTA	2339	ENSG00000168522	NM_001018676
CALM1	801	ENSG00000198668	NM_001166106
HDAC8	55869	ENSG00000147099	NM_001166418
REG1A	5967	ENSG00000115386	NM_002909
HK1	3098	ENSG00000156515	NM_000188
ALDOA	226	ENSG00000149925	NM_000034

FGFR2	2263	ENSG00000066468	NM_000141
NQO2	4835	ENSG00000124588	NM_000904
ATOX1	475	ENSG00000177556	NM_004045
PAK6	56924	ENSG00000137843	NM_001276717
CAT	847	ENSG00000121691	NM_001752
CBR1	873	ENSG00000159228	NM_001286789
DUSP6	1848	ENSG00000139318	NM_001946
NQO1	1728	ENSG00000181019	NM_000903
PRKACA	5566	ENSG00000072062	NM_001304349
PRKACA	5566	ENSG00000288516	NM_001304349
SELP	6403	ENSG00000174175	NM_003005
TNK2	10188	ENSG00000061938	NM_001010938
GPI	2821	ENSG00000105220	NM_000175
GPI	2821	ENSG00000282019	NM_000175
AMD1	262	ENSG00000123505	NM_001033059
CES1	1066	ENSG00000198848	NM_001025194
CES1	1066	ENSG00000262243	NM_001025194
MET	4233	ENSG00000105976	NM_000245
FGFR1	2260	ENSG00000077782	NM_001174063
ALDH2	217	ENSG00000111275	NM_000690
GSTA1	2938	ENSG00000243955	NM_001319059
SYK	6850	ENSG00000165025	NM_001135052
CDK7	1022	ENSG00000134058	NM_001324069
CDK7	1022	ENSG00000277273	NM_001324069
RAB11A	8766	ENSG00000103769	NM_001206836
GLRX	2745	ENSG00000173221	NM_001118890
PIK3CG	5294	ENSG00000105851	NM_001282426
KIT	3815	ENSG00000157404	NM_000222
TTPA	7274	ENSG00000137561	NM_000370
PLEKHA4	57664	ENSG00000105559	NM_001161354
PLA2G10	8399	ENSG00000069764	NM_003561
PLA2G10	8399	ENSG00000276870	NM_003561
DTYMK	1841	ENSG00000168393	NM_001165031
MAN1B1	11253	ENSG00000177239	NM_016219
ARSA	410	ENSG00000100299	NM_000487
GSTT2B	653689	ENSG00000133433	NM_001080843
GSTT2B	653689	ENSG00000278695	NM_001080843
IMPDH1	3614	ENSG00000106348	NM_000883
CTSF	8722	ENSG00000174080	NM_003793
CDK6	1021	ENSG00000105810	NM_001145306
CYP2C9	1559	ENSG00000138109	NM_000771
ADH5	128	ENSG00000197894	NM_000671
ADK	132	ENSG00000156110	NM_001123
PAPSS1	9061	ENSG00000138801	NM_005443

JAK2	3717	ENSG00000096968	NM_001322194
PGF	5228	ENSG00000119630	NM_001207012
HAGH	3029	ENSG00000063854	NM_001040427
PTPN11	5781	ENSG00000179295	NM_001330437
ADAM17	6868	ENSG00000151694	NM_001382777
MMP9	4318	ENSG00000100985	NM_004994
ESR2	2100	ENSG00000140009	NM_001040275
C8G	733	ENSG00000176919	NM_000606
GNPDA1	10007	ENSG00000113552	NM_005471
PDK2	5164	ENSG00000005882	NM_001199898
ARF4	378	ENSG00000168374	NM_001660
JAK3	3718	ENSG00000105639	NM_000215
NR3C1	2908	ENSG00000113580	NM_000176
FABP5	2171	ENSG00000164687	NM_001444
CTSS	1520	ENSG00000163131	NM_001199739
RHOA	387	ENSG00000067560	NM_001313941
NOS2	4843	ENSG00000007171	NM_000625
ZAP70	7535	ENSG00000115085	NM_001079
FABP3	2170	ENSG00000121769	NM_001320996
MMP2	4313	ENSG00000087245	NM_001127891
MAPK12	6300	ENSG00000188130	NM_001303252
MMP1	4312	ENSG00000196611	NM_001145938
FABP6	2172	ENSG00000170231	NM_001040442
LGALS3	3958	ENSG00000131981	NM_001177388
FECH	2235	ENSG00000066926	NM_000140
FDPS	2224	ENSG00000160752	NM_001135821
PTK2	5747	ENSG00000169398	NM_001199649
ARHGAP1	392	ENSG00000175220	NM_004308
IL2	3558	ENSG00000109471	NM_000586
RAF1	5894	ENSG00000132155	NM_001354689
SETD7	80854	ENSG00000145391	NM_001306199
CCL5	6352	ENSG00000271503	NM_001278736
CCL5	6352	ENSG00000274233	NM_001278736
CDC42	998	ENSG00000070831	NM_001039802
HINT1	3094	ENSG00000169567	NM_005340
TEK	7010	ENSG00000120156	NM_000459
GSTM1	2944	ENSG00000134184	NM_000561
MME	4311	ENSG00000196549	NM_000902
NR1I3	9970	ENSG00000143257	NM_001077469
RAB5A	5868	ENSG00000144566	NM_001292048
PRKCQ	5588	ENSG00000065675	NM_001242413
LYZ	4069	ENSG00000090382	NM_000239
PADI4	23569	ENSG00000159339	NM_012387
PADI4	23569	ENSG00000280908	NM_012387

ELANE	1991	ENSG00000197561	NM_001972
ELANE	1991	ENSG00000277571	NM_001972
SULT2B1	6820	ENSG00000088002	NM_004605
GP1BA	2811	ENSG00000185245	NM_000173
GART	2618	ENSG00000159131	NM_000819
GART	2618	ENSG00000262473	NM_000819
HEXB	3074	ENSG00000049860	NM_000521
CTNNA1	1495	ENSG00000044115	NM_001290307
TPH1	7166	ENSG00000129167	NM_004179
PPARA	5465	ENSG00000186951	NM_001001928
MDM2	4193	ENSG00000135679	NM_001145336
SELE	6401	ENSG00000007908	NM_000450
SRM	6723	ENSG00000116649	NM_003132
NMNAT1	64802	ENSG00000173614	NM_001297778
EPHX2	2053	ENSG00000120915	NM_001256482
BIRC7	79444	ENSG00000101197	NM_022161
NR1H4	9971	ENSG00000012504	NM_001206977
GSTA3	2940	ENSG00000174156	NM_000847
S100A9	6280	ENSG00000163220	NM_002965
ACAT1	38	ENSG00000075239	NM_000019
GCDH	2639	ENSG00000105607	NM_000159
CSK	1445	ENSG00000103653	NM_001127190
BHMT	635	ENSG00000145692	NM_001713
AGXT	189	ENSG00000172482	NM_000030
KAT2B	8850	ENSG00000114166	NM_003884
HRAS	3265	ENSG00000174775	NM_001130442
HRAS	3265	ENSG00000276536	NM_001130442

Supplement Table 2. The possible targets of chondrosarcoma.

SYMBOL	ENTREZID	ENSEMBL	REFSEQ
EXT1	2131	ENSG00000182197	NM_000127
NR4A3	8013	ENSG00000119508	NM_006981
TAF15	8148	ENSG00000270647	NM_003487
TAF15	8148	ENSG00000276833	NM_003487
EWSR1	2130	ENSG00000182944	NM_001163285
TFG	10342	ENSG00000114354	NM_001007565
CSAG1	158511	ENSG00000198930	NM_001102576
EXT2	2132	ENSG00000151348	NM_000401
TCF12	6938	ENSG00000140262	NM_001306219
IDH1	3417	ENSG00000138413	NM_001282386
IDH2	3418	ENSG00000182054	NM_001289910
NCOA2	10499	ENSG00000140396	NM_001321703
MMP13	4322	ENSG00000137745	NM_002427
MUC1	4582	ENSG00000185499	NM_001018016
CSAG2	102723547	ENSG00000268902	XM_006724857
PTH1R	5745	ENSG00000160801	NM_000316
SOX9	6662	ENSG00000125398	NM_000346
MMP1	4312	ENSG00000196611	NM_001145938
SYP	6855	ENSG00000102003	NM_003179
S100B	6285	ENSG00000160307	NM_006272
ENO2	2026	ENSG00000111674	NM_001975
RUNX2	860	ENSG00000124813	NM_001015051
VIM	7431	ENSG00000026025	NM_003380
CNMD	11061	ENSG00000136110	NM_001011705
SMARCB1	6598	ENSG00000099956	NM_001007468
SMARCB1	6598	ENSG00000275837	NM_001007468
MTAP	4507	ENSG00000099810	NM_002451
PTHLH	5744	ENSG00000087494	NM_002820
CDK4	1019	ENSG00000135446	NM_000075
SPARC	6678	ENSG00000113140	NM_001309443
S100A1	6271	ENSG00000160678	NM_006271
FGF2	2247	ENSG00000138685	NM_001361665
PDGFRA	5156	ENSG00000134853	NM_001347827
PLAU	5328	ENSG00000122861	NM_001145031
BMP2	650	ENSG00000125845	NM_001200
MET	4233	ENSG00000105976	NM_000245
MMP3	4314	ENSG00000149968	NM_002422
MDM2	4193	ENSG00000135679	NM_001145336
MYOG	4656	ENSG00000122180	NM_002479
GFAP	2670	ENSG00000131095	NM_001131019
SERPINA3	12	ENSG00000196136	NM_001085

BCAR4	400500	ENSG00000262117	NR_024049
CCN2	1490	ENSG00000118523	NM_001901
COL10A1	1300	ENSG00000123500	NM_000493
FGFR3	2261	ENSG00000068078	NM_000142
ADAMTS4	9507	ENSG00000158859	NM_001320336
FUS	2521	ENSG00000089280	NM_001010850
NFATC2	4773	ENSG00000101096	NM_001136021
COL11A2	1302	ENSG00000204248	NM_001163771
COL11A2	1302	ENSG00000223699	NM_001163771
COL11A2	1302	ENSG00000232541	NM_001163771
COL11A2	1302	ENSG00000235708	NM_001163771
COL11A2	1302	ENSG00000230930	NM_001163771
COL11A2	1302	ENSG00000206290	NM_001163771
COL11A2	1302	ENSG00000227801	NM_001163771
HEY1	23462	ENSG00000164683	NM_001040708
DES	1674	ENSG00000175084	NM_001382708
KIT	3815	ENSG00000157404	NM_000222
ETV4	2118	ENSG00000175832	NM_001079675
SS18	6760	ENSG00000141380	NM_001007559
PLAG1	5324	ENSG00000181690	NM_001114634
ETV1	2115	ENSG00000006468	NM_001163147
PATZ1	23598	ENSG00000100105	NM_014323
ZNF444	55311	ENSG00000167685	NM_001253792
DUX4	100288687	ENSG00000260596	NM_001205218
DUX4	100288687	ENSG00000283949	NM_001205218
DUX4L7	653543	ENSG00000281652	NM_001127387
DUX4L8	26583	ENSG00000281720	NM_012147
DUX4L1	22947	ENSG00000280757	NM_033178
CD99	4267	ENSG00000002586	NM_001122898
COL2A1	1280	ENSG00000139219	NM_001844
DDIT3	1649	ENSG00000175197	NM_001195053
MAPT	4137	ENSG00000186868	NM_001123066
MAPT	4137	ENSG00000276155	NM_001123066
MAPT	4137	ENSG00000277956	NM_001123066
VEGFA	7422	ENSG00000112715	NM_001025366
NKX2-2	4821	ENSG00000125820	NM_002509
CDKN2A	1029	ENSG00000147889	NM_000077
CDKN2B	1030	ENSG00000147883	NM_004936
MMP2	4313	ENSG00000087245	NM_001127891
TLE1	7088	ENSG00000196781	NM_001303103
SSX1	6756	ENSG00000126752	NM_001278691
NKX3-1	4824	ENSG00000167034	NM_001256339
IK	3550	ENSG00000113141	NM_006083
WT1	7490	ENSG00000184937	NM_000378

PBX1	5087	ENSG00000185630	NM_001204961
NR4A1	3164	ENSG00000123358	NM_001202233
CD34	947	ENSG00000174059	NM_001025109
PBX3	5090	ENSG00000167081	NM_001134778
CREB3L2	64764	ENSG00000182158	NM_001253775
CCNB3	85417	ENSG00000147082	NM_033031
SSX2	6757	ENSG00000241476	NM_001278697
PCDHA8	56140	ENSG00000204962	NM_018911
ZMAT2	153527	ENSG00000146007	NM_144723
MAPK1	5594	ENSG00000100030	NM_002745
MYOD1	4654	ENSG00000129152	NM_002478
VEGFC	7424	ENSG00000150630	NM_005429
H3-3A	3020	ENSG00000163041	NM_001379043
PRKCA	5578	ENSG00000154229	NM_002737
KRT7	3855	ENSG00000135480	NM_005556
CD68	968	ENSG00000129226	NM_001040059
CDX1	1044	ENSG00000113722	NM_001804
IRF2BP2	359948	ENSG00000168264	NM_001077397
COMP	1311	ENSG00000105664	NM_000095
MMP9	4318	ENSG00000100985	NM_004994
YEATS2	55689	ENSG00000163872	NM_001351369
IDH3G	3421	ENSG00000067829	NM_004135
H3-3B	3021	ENSG00000132475	NM_005324
CSAG3	389903	ENSG00000268916	NM_001129826
CLCN3	1182	ENSG00000109572	NM_001243372
ITGAV	3685	ENSG00000138448	NM_001144999
ITGB3	3690	ENSG00000259207	NM_000212
PLGLB1	5343	ENSG00000183281	NM_001032392
BCL2L1	598	ENSG00000171552	NM_001191
TP53	7157	ENSG00000141510	NM_000546
PTK2	5747	ENSG00000169398	NM_001199649
CDKN1A	1026	ENSG00000124762	NM_000389
H2AC18	8337	ENSG00000203812	NM_003516
IL1B	3553	ENSG00000125538	NM_000576
AREG	374	ENSG00000109321	NM_001657
ITGB1	3688	ENSG00000150093	NM_002211
IHH	3549	ENSG00000163501	NM_002181
CEACAM3	1084	ENSG00000170956	NM_001277163
BCL2	596	ENSG00000171791	NM_000633
CXCR4	7852	ENSG00000121966	NM_001008540
NFKB1	4790	ENSG00000109320	NM_001165412
STAT3	6774	ENSG00000168610	NM_001369512
HIF1A	3091	ENSG00000100644	NM_001243084
BDNF	627	ENSG00000176697	NM_001143805

ETF1	2107	ENSG00000120705	NM_001256302
SEC31A	22872	ENSG00000138674	NM_001077206
VPS72	6944	ENSG00000163159	NM_001271087
MIR204	406987	ENSG00000207935	NR_029621
EGFR	1956	ENSG00000146648	NM_001346897
MIR145	406937	ENSG00000276365	NR_029686
IL6	3569	ENSG00000136244	NM_000600
TET2	54790	ENSG00000168769	NM_001127208
PPARG	5468	ENSG00000132170	NM_001330615
RB1	5925	ENSG00000139687	NM_000321
TIMP2	7077	ENSG00000035862	NM_003255
MIR30A	407029	ENSG00000207827	NR_029504
XIAP	331	ENSG00000101966	NM_001167
PTGS2	5743	ENSG00000073756	NM_000963
MMP14	4323	ENSG00000157227	NM_004995
TNFSF11	8600	ENSG00000120659	NM_003701
CSAG4	100130935	ENSG00000242599	NM_001025306
TNFRSF11A	8792	ENSG00000141655	NM_001270949
NAB2	4665	ENSG00000166886	NM_001330305
PPCS	79717	ENSG00000127125	NM_001077447
METTL14	57721	ENSG00000145388	NM_020961
AMER1	139285	ENSG00000184675	NM_152424
YTHDC1	91746	ENSG00000083896	NM_001031732
YTHDC1	91746	ENSG00000275272	NM_001031732
ALKBH5	54890	ENSG00000091542	NM_017758
RAB6C	84084	ENSG00000222014	NM_032144
H3C4	8351	ENSG00000197409	NM_001376937
H3C3	8352	ENSG00000287080	NM_003531
H3C6	8353	ENSG00000274750	NM_001381999
RETN	56729	ENSG00000104918	NM_001193374
TERT	7015	ENSG00000164362	NM_001193376
TGFB1	7040	ENSG00000105329	NM_000660
CXCL12	6387	ENSG00000107562	NM_000609
ADAMTS16	170690	ENSG00000145536	NM_139056
PDCD5	9141	ENSG00000105185	NM_004708
TBXT	6862	ENSG00000164458	NM_001270484
NAMPT	10135	ENSG00000105835	NM_005746
BAX	581	ENSG00000087088	NM_001291428
SIX3	6496	ENSG00000138083	NM_005413
ADAMTS9	56999	ENSG00000163638	NM_001318781
MAPK3	5595	ENSG00000102882	NM_001040056
KDM4C	23081	ENSG00000107077	NM_001146694
TNF	7124	ENSG00000232810	NM_000594
TNF	7124	ENSG00000228978	NM_000594

TNF	7124	ENSG00000230108	NM_000594
TNF	7124	ENSG00000223952	NM_000594
TNF	7124	ENSG00000228321	NM_000594
TNF	7124	ENSG00000228849	NM_000594
TNF	7124	ENSG00000204490	NM_000594
TNF	7124	ENSG00000206439	NM_000594
TNC	3371	ENSG00000041982	NM_002160
IFI27	3429	ENSG00000165949	NM_001130080
IFI27	3429	ENSG00000275214	NM_001130080
CASP3	836	ENSG00000164305	NM_001354777
IGF1	3479	ENSG00000017427	NM_000618
TGFB2	7042	ENSG00000092969	NM_001135599
CD274	29126	ENSG00000120217	NM_001267706
PDPN	10630	ENSG00000162493	NM_001006624
EZR	7430	ENSG00000092820	NM_001111077
PIK3CG	5294	ENSG00000105851	NM_001282426
ITGA8	8516	ENSG00000077943	NM_001291494
VNN2	8875	ENSG00000112303	NM_001242350
MCHR2	84539	ENSG00000152034	NM_001040179
MAML1	9794	ENSG00000161021	NM_014757
MAML1	9794	ENSG00000283780	NM_014757
ADHFE1	137872	ENSG00000147576	NM_144650
FBXW5	54461	ENSG00000159069	NM_018998
OGFOD1	55239	ENSG00000087263	NM_001324357
JMJD4	65094	ENSG00000081692	NM_001161465
PLGLB2	5342	ENSG00000125551	NM_002665
ZNF80	7634	ENSG00000174255	NM_007136
MIR33B	693120	ENSG00000207839	NR_030361
ACTC1	70	ENSG00000159251	NM_005159
GNAS	2778	ENSG00000087460	NM_000516
ATRX	546	ENSG00000085224	NM_000489
CEP57	9702	ENSG00000166037	NM_001243776
GPR108	56927	ENSG00000125734	NM_001080452
CCDC26	137196	ENSG00000229140	NR_130917
NFKBIA	4792	ENSG00000100906	NM_020529
ENG	2022	ENSG00000106991	NM_000118
MTOR	2475	ENSG00000198793	NM_001386500
ACAN	176	ENSG00000157766	NM_001135
CCND1	595	ENSG00000110092	NM_053056
MIR491	574444	ENSG00000207609	NR_030166
SP1	6667	ENSG00000185591	NM_001251825
EPAS1	2034	ENSG00000116016	NM_001430
SIRT1	23411	ENSG00000096717	NM_001142498
ADIPOR1	51094	ENSG00000159346	NM_001290553

ADIPOR2	79602	ENSG00000006831	NM_001375363
ADIPOR2	79602	ENSG00000285070	NM_001375363
MIR181A1	406995	ENSG00000207759	NR_029626
NFATC1	4772	ENSG00000131196	NM_001278669
HDAC4	9759	ENSG00000068024	NM_001378414
SDC2	6383	ENSG00000169439	NM_002998
MYC	4609	ENSG00000136997	NM_001354870
MIR199A1	406976	ENSG00000207752	NR_029586
FGFR1	2260	ENSG00000077782	NM_001174063
ESRRB	2103	ENSG00000119715	NM_001379180
HSPA5	3309	ENSG00000044574	NM_005347
CXCL8	3576	ENSG00000169429	NM_000584
MMP7	4316	ENSG00000137673	NM_002423
CCL2	6347	ENSG00000108691	NM_002982
RAF1	5894	ENSG00000132155	NM_001354689
ITGB5	3693	ENSG00000082781	NM_001354764
HNF1A	6927	ENSG00000135100	NM_000545
E2F1	1869	ENSG00000101412	NM_005225
CCL5	6352	ENSG00000271503	NM_001278736
CCL5	6352	ENSG00000274233	NM_001278736
PEG10	23089	ENSG00000242265	NM_001040152
MIR27B	407019	ENSG00000207864	NR_029665
CEBPB	1051	ENSG00000172216	NM_001285878
MIR143	406935	ENSG00000284182	NR_029684
PDGFRB	5159	ENSG00000113721	NM_001355016
SRC	6714	ENSG00000197122	NM_005417
DKK1	22943	ENSG00000107984	NM_012242
CCN3	4856	ENSG00000136999	NM_002514
CD44	960	ENSG00000026508	NM_000610
ICAM1	3383	ENSG00000090339	NM_000201
PRKAA1	5562	ENSG00000132356	NM_001355028
LEP	3952	ENSG00000174697	NM_000230
HSPG2	3339	ENSG00000142798	NM_001291860
ISG20	3669	ENSG00000172183	NM_001303233
CCN6	8838	ENSG00000112761	NM_003880
IKBKB	3551	ENSG00000104365	NM_001190720
BDKRB1	623	ENSG00000100739	NM_000710
CTSB	1508	ENSG00000164733	NM_001317237
CTSB	1508	ENSG00000285132	NM_001317237
ITGA6	3655	ENSG00000091409	NM_000210
RELA	5970	ENSG00000173039	NM_001145138
ITGA5	3678	ENSG00000161638	NM_002205
ITGA2	3673	ENSG00000164171	NM_002203
ADAMTS1	9510	ENSG00000154734	NM_006988

RUNX3	864	ENSG00000020633	NM_001031680
IL11	3589	ENSG00000095752	NM_000641
PRKCD	5580	ENSG00000163932	NM_001316327
ABL1	25	ENSG00000097007	NM_005157
MAPK14	1432	ENSG00000112062	NM_001315
PARP1	142	ENSG00000143799	NM_001618
EDN1	1906	ENSG00000078401	NM_001168319
IGF2	3481	ENSG00000167244	NM_000612
EFNA5	1946	ENSG00000184349	NM_001962
TIMP3	7078	ENSG00000100234	NM_000362
MAPK10	5602	ENSG00000109339	NM_001318067
MTHFR	4524	ENSG00000177000	NM_001330358
JUN	3725	ENSG00000177606	NM_002228
ETV6	2120	ENSG00000139083	NM_001987
GLI1	2735	ENSG00000111087	NM_001160045
ZBTB7A	51341	ENSG00000178951	NM_001317990
PDGFB	5155	ENSG00000100311	NM_002608
CHGA	1113	ENSG00000100604	NM_001275
CHGA	1113	ENSG00000276781	NM_001275
PARVA	55742	ENSG00000197702	NM_018222
MMP12	4321	ENSG00000262406	NM_002426
ADAMTS5	11096	ENSG00000154736	NM_007038
IL1A	3552	ENSG00000115008	NM_000575
LGALS1	3956	ENSG00000100097	NM_002305
CYCS	54205	ENSG00000172115	NM_018947
PLAGL1	5325	ENSG00000118495	NM_001080951
FH	2271	ENSG00000091483	NM_000143
IDH3B	3420	ENSG00000101365	NM_001258384
D2HGDH	728294	ENSG00000180902	NM_001287249
AGGF1	55109	ENSG00000164252	NM_018046
L2HGDH	79944	ENSG00000087299	NM_024884
PDCD1	5133	ENSG00000188389	NM_005018
PDCD1	5133	ENSG00000276977	NM_005018
ESR2	2100	ENSG00000140009	NM_001040275
CDH1	999	ENSG00000039068	NM_001317184
MIR381	494330	ENSG00000199020	NR_029873
FOS	2353	ENSG00000170345	NM_005252
TGFBR1	7046	ENSG00000106799	NM_001130916
SATB2	23314	ENSG00000119042	NM_001172509
BAG3	9531	ENSG00000151929	NM_004281
MMP19	4327	ENSG00000123342	NM_001032360
INSM1	3642	ENSG00000173404	NM_002196
TGFBR2	7048	ENSG00000163513	NM_001024847
TP63	8626	ENSG00000073282	NM_001114978

TGFB3	7043	ENSG00000119699	NM_001329938
NCAM1	4684	ENSG00000149294	NM_000615
BMPR2	659	ENSG00000204217	NM_001204
JUP	3728	ENSG00000173801	NM_001352773
HSPA1A	3303	ENSG00000204389	NM_005345
HSPA1A	3303	ENSG00000235941	NM_005345
HSPA1A	3303	ENSG00000234475	NM_005345
HSPA1A	3303	ENSG00000215328	NM_005345
HSPA1A	3303	ENSG00000237724	NM_005345
CSPG4	1464	ENSG00000173546	NM_001897
MKI67	4288	ENSG00000148773	NM_001145966
ID1	3397	ENSG00000125968	NM_002165
MIR206	406989	ENSG00000207604	NR_029713
AURKA	6790	ENSG00000087586	NM_001323303
CTSK	1513	ENSG00000143387	NM_000396
PRMT1	3276	ENSG00000126457	NM_001207042
CCN4	8840	ENSG00000104415	NM_001204869
LDHA	3939	ENSG00000134333	NM_001135239
LDHA	3939	ENSG00000288299	NM_001135239
AURKB	9212	ENSG00000178999	NM_001256834
HDAC9	9734	ENSG00000048052	NM_001204144
GADD45B	4616	ENSG00000099860	NM_015675
MIR125B2	406912	ENSG00000207863	NR_029694
MIR125B1	406911	ENSG00000207971	NR_029671
MIR494	574452	ENSG00000194717	NR_030174
TEC	7006	ENSG00000135605	NM_003215
BGLAP	632	ENSG00000242252	NM_199173
POMGNT2	84892	ENSG00000144647	NM_032806
ABCB1	5243	ENSG00000085563	NM_000927
CDC25A	993	ENSG00000164045	NM_001789
YAP1	10413	ENSG00000137693	NM_001130145
PRKAB1	5564	ENSG00000111725	NM_006253
SOX4	6659	ENSG00000124766	NM_003107
SMAD1	4086	ENSG00000170365	NM_001003688
SFRP5	6425	ENSG00000120057	NM_003015
PRAME	23532	ENSG00000185686	NM_001291715
PRAME	23532	ENSG00000275013	NM_001291715
CTAG2	30848	ENSG00000126890	NM_020994
MIR497	574456	ENSG00000284027	NR_030178
MMP8	4317	ENSG00000118113	NM_001304441
MYCN	4613	ENSG00000134323	NM_001293228
NOS2	4843	ENSG00000007171	NM_000625
CTSL	1514	ENSG00000135047	NM_001257971
BECN1	8678	ENSG00000126581	NM_001313998

THY1	7070	ENSG00000154096	NM_001311160
ADAM28	10863	ENSG00000042980	NM_001304351
NAPRT	93100	ENSG00000147813	NM_001286829
NAPRT	93100	ENSG00000278488	NM_001286829
TOMM20	9804	ENSG00000173726	NM_014765
MIR342	442909	ENSG00000199082	NR_029888
MIR186	406962	ENSG00000207721	NR_029707
BRAF	673	ENSG00000157764	NM_001354609
HDAC6	10013	ENSG00000094631	NM_001321225
CASP9	842	ENSG00000132906	NM_001229
AMACR	23600	ENSG00000242110	NM_001167595
CCR7	1236	ENSG00000126353	NM_001301714
AGER	177	ENSG00000204305	NM_001136
AGER	177	ENSG00000231268	NM_001136
AGER	177	ENSG00000229058	NM_001136
AGER	177	ENSG00000237405	NM_001136
AGER	177	ENSG00000230514	NM_001136
AGER	177	ENSG00000206320	NM_001136
AGER	177	ENSG00000234729	NM_001136
XRCC5	7520	ENSG00000079246	NM_021141
SNAI2	6591	ENSG00000019549	NM_003068
CCL21	6366	ENSG00000137077	NM_002989
PTGER1	5731	ENSG00000160951	NM_000955
TNFRSF8	943	ENSG00000120949	NM_001243
RGS16	6004	ENSG00000143333	NM_002928
MIR101-1	406893	ENSG00000199135	NR_029516
CD36	948	ENSG00000135218	NM_000072
FGF1	2246	ENSG00000113578	NM_000800
TIMP1	7076	ENSG00000102265	NM_003254
HGF	3082	ENSG00000019991	NM_000601
SERPINE1	5054	ENSG00000106366	NM_000602
EIF2AK3	9451	ENSG00000172071	NM_001313915
PLAT	5327	ENSG00000104368	NM_000930
FLI1	2313	ENSG00000151702	NM_001167681
MAP3K5	4217	ENSG00000197442	NM_005923
SETD2	29072	ENSG00000181555	NM_001349370
MYH11	4629	ENSG00000133392	NM_001040113
MYH11	4629	ENSG00000276480	NM_001040113
NCOA3	8202	ENSG00000124151	NM_001174087
ADIPOQ	9370	ENSG00000181092	NM_001177800
KRT19	3880	ENSG00000171345	NM_002276
THBS1	7057	ENSG00000137801	NM_003246
NCOA1	8648	ENSG00000084676	NM_001362950
POSTN	10631	ENSG00000133110	NM_001135934

MCM6	4175	ENSG00000076003	NM_005915
IGF2BP3	10643	ENSG00000136231	NM_006547
ATG12	9140	ENSG00000145782	NM_001277783
MAGEA3	4102	ENSG00000221867	NM_005362
MIR100	406892	ENSG00000207994	NR_029515
MIR200B	406984	ENSG00000207730	NR_029639
MIR199A2	406977	ENSG00000208024	NR_029618
MIR129-2	406918	ENSG00000199077	NR_029697
MIR454	768216	ENSG00000211514	NR_030411
MIR129-1	406917	ENSG00000207705	NR_029596
MIR519D	574480	ENSG00000207981	NR_030202
PTPN11	5781	ENSG00000179295	NM_001330437
HSP90AA1	3320	ENSG00000080824	NM_001017963
ACP5	54	ENSG00000102575	NM_001111034
PTH	5741	ENSG00000152266	NM_000315
TDP1	55775	ENSG00000042088	NM_001008744
GSTA3	2940	ENSG00000174156	NM_000847
UBE2R2	54926	ENSG00000107341	NM_017811
PTGR1	22949	ENSG00000106853	NM_001146108
MAML3	55534	ENSG00000196782	NM_018717
RCN3	57333	ENSG00000142552	NM_020650
IL10	3586	ENSG00000136634	NM_000572
BMP6	654	ENSG00000153162	NM_001718
CAPN1	823	ENSG00000014216	NM_001198868
DAPK1	1612	ENSG00000196730	NM_001288729
CAPN2	824	ENSG00000162909	NM_001146068
CD40LG	959	ENSG00000102245	NM_000074
HSPD1	3329	ENSG00000144381	NM_002156
CRP	1401	ENSG00000132693	NM_000567
VEGFB	7423	ENSG00000173511	NM_001243733
KLF6	1316	ENSG00000067082	NM_001008490
FHIT	2272	ENSG00000189283	NM_001166243
RPS6	6194	ENSG00000137154	NM_001010
MUC5B	727897	ENSG00000117983	NM_002458
PPFIBP1	8496	ENSG00000110841	NM_001198915
CCL3	6348	ENSG00000277632	NM_002983
CCL3	6348	ENSG00000278567	NM_002983
CCL3	6348	ENSG00000274221	NM_002983
ETV5	2119	ENSG00000244405	NM_004454
VEGFD	2277	ENSG00000165197	NM_004469
CCN1	3491	ENSG00000142871	NM_001554
SNHG6	641638	ENSG00000245910	NR_002599
MIR624	693209	ENSG00000207952	NR_030354
CDK2	1017	ENSG00000123374	NM_001290230

CHUK	1147	ENSG00000213341	NM_001278
MIF	4282	ENSG00000240972	NM_002415
MIF	4282	ENSG00000276701	NM_002415
IRS1	3667	ENSG00000169047	NM_005544
GATA1	2623	ENSG00000102145	NM_002049
CCNB1	891	ENSG00000134057	NM_001354844
SPP1	6696	ENSG00000118785	NM_000582
HSP90B1	7184	ENSG00000166598	NM_003299
HMGB1	3146	ENSG00000189403	NM_001313892
FSCN1	6624	ENSG00000075618	NM_003088
SYNM	23336	ENSG00000182253	NM_015286
CTSD	1509	ENSG00000117984	NM_001909
HRAS	3265	ENSG00000174775	NM_001130442
HRAS	3265	ENSG00000276536	NM_001130442
COL1A2	1278	ENSG00000164692	NM_000089
PIK3CB	5291	ENSG00000051382	NM_001256045
TNFRSF11B	4982	ENSG00000164761	NM_002546
KNG1	3827	ENSG00000113889	NM_000893
F2R	2149	ENSG00000181104	NM_001311313
F2RL3	9002	ENSG00000127533	NM_003950
BDKRB2	624	ENSG00000168398	NM_000623
TBX3	6926	ENSG00000135111	NM_005996
SERPINB2	5055	ENSG00000197632	NM_001143818
PRPH	5630	ENSG00000135406	NM_006262
HS3ST1	9957	ENSG00000002587	NM_005114
APOBEC3B	9582	ENSG00000179750	NM_001270411
HS3ST2	9956	ENSG00000122254	NM_006043
IGF1R	3480	ENSG00000140443	NM_000875
RET	5979	ENSG00000165731	NM_000323
AKT1	207	ENSG00000142208	NM_001014431
SMO	6608	ENSG00000128602	NM_005631
MYB	4602	ENSG00000118513	NM_001130172
PTCH1	5727	ENSG00000185920	NM_000264
KDM1A	23028	ENSG00000004487	NM_001009999
FGR	2268	ENSG00000000938	NM_001042729
ILK	3611	ENSG00000166333	NM_001014794
TGFA	7039	ENSG00000163235	NM_001099691
GFM1	85476	ENSG00000168827	NM_001308164
GAB1	2549	ENSG00000109458	NM_002039
TIAM1	7074	ENSG00000156299	NM_001353684
FGF3	2248	ENSG00000186895	NM_005247
SPINT2	10653	ENSG00000167642	NM_001166103
PDGFA	5154	ENSG00000197461	NM_002607
LUM	4060	ENSG00000139329	NM_002345

CDC37	11140	ENSG00000105401	NM_007065
FERMT2	10979	ENSG00000073712	NM_001134999
PARVB	29780	ENSG00000188677	NM_001003828
FBLIM1	54751	ENSG00000162458	NM_001024215
MIR518B	574474	ENSG00000207862	NR_030196

Supplement Table 3. The potential targets associated with SA against chondrosarcoma.

SYMBOL	ENTREZID	ENSEMBL	REFSEQ
CASP3	836	ENSG00000164305	NM_001354777
BMP2	650	ENSG00000125845	NM_001200
CDK2	1017	ENSG00000123374	NM_001290230
MAPK10	5602	ENSG00000109339	NM_001318067
CTSD	1509	ENSG00000117984	NM_001909
TGFBR2	7048	ENSG00000163513	NM_001024847
PLAU	5328	ENSG00000122861	NM_001145031
IGF1R	3480	ENSG00000140443	NM_000875
SRC	6714	ENSG00000197122	NM_005417
MAPK1	5594	ENSG00000100030	NM_002745
MMP8	4317	ENSG00000118113	NM_001304441
MMP3	4314	ENSG00000149968	NM_002422
MTAP	4507	ENSG00000099810	NM_002451
MMP13	4322	ENSG00000137745	NM_002427
EGFR	1956	ENSG00000146648	NM_001346897
MAPK14	1432	ENSG00000112062	NM_001315
CTSB	1508	ENSG00000164733	NM_001317237
CTSB	1508	ENSG00000285132	NM_001317237
HSP90AA1	3320	ENSG00000080824	NM_001017963
TGFBR1	7046	ENSG00000106799	NM_001130916
AKT1	207	ENSG00000142208	NM_001014431
MMP12	4321	ENSG00000262406	NM_002426
FGF1	2246	ENSG00000113578	NM_000800
PARP1	142	ENSG00000143799	NM_001618
CTSK	1513	ENSG00000143387	NM_000396
FHIT	2272	ENSG00000189283	NM_001166243
PPARG	5468	ENSG00000132170	NM_001330615
MIF	4282	ENSG00000240972	NM_002415
MIF	4282	ENSG00000276701	NM_002415
DAPK1	1612	ENSG00000196730	NM_001288729
ISG20	3669	ENSG00000172183	NM_001303233
MMP7	4316	ENSG00000137673	NM_002423
XIAP	331	ENSG00000101966	NM_001167
MET	4233	ENSG00000105976	NM_000245
FGFR1	2260	ENSG00000077782	NM_001174063
PIK3CG	5294	ENSG00000105851	NM_001282426
KIT	3815	ENSG00000157404	NM_000222
PTPN11	5781	ENSG00000179295	NM_001330437
MMP9	4318	ENSG00000100985	NM_004994
ESR2	2100	ENSG00000140009	NM_001040275
NOS2	4843	ENSG00000007171	NM_000625

MMP2	4313	ENSG00000087245	NM_001127891
MMP1	4312	ENSG00000196611	NM_001145938
PTK2	5747	ENSG00000169398	NM_001199649
RAF1	5894	ENSG00000132155	NM_001354689
CCL5	6352	ENSG00000271503	NM_001278736
CCL5	6352	ENSG00000274233	NM_001278736
MDM2	4193	ENSG00000135679	NM_001145336
GSTA3	2940	ENSG00000174156	NM_000847
HRAS	3265	ENSG00000174775	NM_001130442
HRAS	3265	ENSG00000276536	NM_001130442
